# Supervised machine learning aided behavior classification in pigeons

**DOI:** 10.3758/s13428-022-01881-w

**Published:** 2022-06-14

**Authors:** Neslihan Wittek, Kevin Wittek, Christopher Keibel, Onur Güntürkün

**Affiliations:** 1 Faculty of Psychology, Department of Biopsychology, Institute of Cognitive Neuroscience, Ruhr University Bochum, Universitätsstraße 150, 44801 Bochum, Germany; 2 Faculty of Mathematics, Computer Science and Natural Sciences, Department of Computer Science, RWTH Aachen University, Aachen, Germany; 3 Institute for Internet Security, Westphalian University of Applied Sciences, Gelsenkirchen, Germany

**Keywords:** Actions, Sequence analysis, Ethogram, DeepLabCut, Birds, Deep learning

## Abstract

Manual behavioral observations have been applied in both environment and laboratory experiments in order to analyze and quantify animal movement and behavior. Although these observations contributed tremendously to ecological and neuroscientific disciplines, there have been challenges and disadvantages following in their footsteps. They are not only time-consuming, labor-intensive, and error-prone but they can also be subjective, which induces further difficulties in reproducing the results. Therefore, there is an ongoing endeavor towards automated behavioral analysis, which has also paved the way for open-source software approaches. Even though these approaches theoretically can be applied to different animal groups, the current applications are mostly focused on mammals, especially rodents. However, extending those applications to other vertebrates, such as birds, is advisable not only for extending species-specific knowledge but also for contributing to the larger evolutionary picture and the role of behavior within. Here we present an open-source software package as a possible initiation of bird behavior classification. It can analyze pose-estimation data generated by established deep-learning-based pose-estimation tools such as DeepLabCut for building supervised machine learning predictive classifiers for pigeon behaviors, which can be broadened to support other bird species as well. We show that by training different machine learning and deep learning architectures using multivariate time series data as input, an F1 score of 0.874 can be achieved for a set of seven distinct behaviors. In addition, an algorithm for further tuning the bias of the predictions towards either precision or recall is introduced, which allows tailoring the classifier to specific needs.

**Supplementary Information:**

The online version contains supplementary material available at 10.3758/s13428-022-01881-w.

## Introduction

Neuroscience has taken a breathtaking ascent within just a few decades (Cobb, 2020). Despite countless success stories at the molecular, cellular, and clinical levels, the explanation of behavior by reverse engineering of neural components or by other bottom-up means has fallen short (Peebles & Cooper, 2015; Jonas & Kording, 2017). Instead, behavior itself has also to be analyzed with the same painstaking accuracy as done in other neuroscientific fields (Tinbergen, 1963; Krakauer et al., 2017). As concisely phrased by Jerry Hirsh, “Nothing in neurobiology makes sense, except in the light of behavior” (Hirsh, 1986).

The detailed analysis of animal behavior also paved the way to modern experimental psychology and still greatly contributes to various psychological insights (Thorndike, 1898; Pavlov, 1927; Skinner, 1938; Kilian et al., 2003; Vallortigara et al., 2005; Zentall et al., 2013; Du et al., 2016; Anselme, 2021). Ecology-driven research fields are particularly interested in the evolutionary roots of animal behavior, which can be affected by external factors such as limitations of nutrients, territories, or mates (Brown, 1969; Baker, 1972; Gill & Wolf, 1975, Aragón et al., 2003; Arak, 1983; Bailey, 2003; Brown et al., 2006; Bentsen et al., 2006; Anselme & Güntürkün, 2019), whereas experimental and molecular biology combine their methods with behavioral observations to investigate medical conditions such as Parkinson’s disease and early life stress (Kravitz et al., 2010; Mundorf et al., 2020). In addition, neuroscientists co-analyze behavioral paradigms in their experimental designs to identify the functional relevance of their neurobiological findings (Miri et al., 2017; Caggiano et al., 2018; Branco & Redgrave, 2020; Packheiser et al., 2021).

In order to quantify animal movement and behavior, both natural-habitat and laboratory experiments have continuously benefitted from on-site manual behavioral observations (von Frisch, 1967; Lindburg, 1969; Gallup, 1970; Calhoun, 1970; Altmann, 1974; Anschel & Talmage-Riggs, 1977; Pepperberg et al., 1995; Pollok et al., 2000; Reiss & Marino, 2001; Dally et al., 2006; Prior et al., 2008). Despite all those pioneering contributions, certain challenges and disadvantages follow in the footsteps of manual behavioral observations: They are not only time-consuming and labor-intensive but also have a grain of subjectivity, which might lead to difficulties in reproducing the experiments (Dell et al., 2014). The issues resulting from subjectivity may be mitigated by using camera video recording systems. Unlike a direct observation, a video recording ensures the capture of complete (within the captured dimensions) and detailed behavioral patterns during the observation period (Tosi et al., 2006). However, analyzing video recordings using a traditional approach involving pencil, paper, and stopwatch is time consuming as well. Furthermore, missed detections are still possible due to fluctuating attention of the observer (Anderson & Perona, 2014; Gomez-Marin et al.,^2014^; Arac et al., 2019).

Besides all these challenges in analyzing behavior, it should not be forgotten that behavior in itself is a complex, dynamic, and multi-dimensional domain (Gomez-Marin et al., 2014), which makes exploring innovative approaches a sensible strategy. At this stage, the recent technological developments in the field of computer vision in conjunction with a newfound interest in artificial intelligence applications have been supporting researchers: Less time and effort is needed to produce precise datasets of animal movement and behavior and the tracking of the animals can be done automatically, which minimizes the amount of human labor and the potential for missed detections (Dell et al., 2014; Bello-Arroyo et al.,2018). Consequently, researchers have been working with different commercial-proprietary and open-source software for the automated analysis of animal behavior with a focus on a particular animal model or different sets of animal groups (commercial-proprietary- EthoVision: Noldus et al., 2001, VideoTrack: ViewPoint Behavior Technology, ANY-maze: Stoelting, Wood Dale, IL, USA; open-source- SwisTrack: Lochmatter et al., 2008; Ethowatcher: Crispim Junior et al., 2012; JAABA: Kabra et al., 2013; idTracker: Pérez-Escudero et al., 2014; DeepLabCut: Mathis et al., 2018; MouBeAT: Bello-Arroyo et al., 2018; UMATracker: Yamanaka & Takeuchi, 2018; Tracktor: Sridhar et al., 2019; TRex: Walter & Cousin, 2020). These software products have not only provided the foundation for quantitative and precise results like velocity, body-orientation, trajectory, and time spent in a particular area (Evans et al., 2015; Singh et al., 2016; Dankert et al., 2009; Luyten et al., 2014, Wittek et al., 2021), but have also established the groundwork for automated analysis of measuring complex behaviors such as anxiety, stress, aggressiveness, risk assessment, shoaling, and spatial learning in different animal groups (Rodríguez et al., 2004; Choy et al., 2012; Piato et al., 2011; Green et al., 2012; Miller & Gerlai, 2012; Nema et al., 2016; Peng et al., 2016; Mazur-Milecka & Ruminski, 2017; Mundorf et al., 2020). Extending those applications to birds is advisable not only for extending the species-specific knowledge but also for contributing to the bigger picture of the evolutionary process. Most importantly, studies on pigeons have a long tradition in experimental psychology and importantly have contributed to insights about learning and memory (Vaughan & Greene, 1984; Troje et al., 1999; Fagot & Cook, 2006; Pearce et al., 2008; Wilzeck et al., 2010; Rose et al., 2009; Scarf et al., 2016; Güntürkün et al., 2018; Packheiser et al., 2019). However, so far, the application of these automated analyses on birds is still limited. But there is a different species, which lends itself to a closer investigation with regards to automated behavior classification: *Homo sapiens*. Existing applications from industry and academia in the domain of human–computer interaction and computer vision have spun up a vast array of literature, mathematical models, and software approaches for human activity recognition, which should be further investigated in order to establish a baseline.

The increasingly large amount of data acquired by different technical devices and sensors, some of them ubiquitous to today’s human life (e.g., “smart devices” such as phones and watches), resulted in an explorative renaissance of machine learning methods by leveraging image- as well as sensor-data (raw or pre-processed) for human activity recognition. Various classification algorithms such as support-vector machine, hidden Markov model, decision tree, random forest, k-nearest neighbors, logistic regression, and stochastic gradient descent have been used to successfully analyze and classify human physical activity (Mannini & Sabatini, 2010; Anguita et al., 2012; Paul & George, 2015; Kolekar & Dash, 2016; Xu et al., 2017; Nematallah et al., 2019; Baldominos et al., 2019). In addition to these traditional machine learning methods, the emergence and widespread availability of new hardware allowing the use of deep learning architectures has motivated a tendency towards using deep learning approaches for human activity recognition as well. These include recurrent neural networks (RNNs) (Murakami & Taguchi, 1991; Murad & Pyun, 2017; Carfi et al., 2018; Koch et al., 2019), long short-term memory (LSTM) (Chen et al., 2016; Singh et al., 2017; Pienaar & Malekian, 2019) and convolutional neural networks (CNN) (Wang et al., 2019; Lee et al., 2017; Gholamrezaii & Taghi Almodarresi, 2019; Naqvi et al., 2020; Cruciani et al., 2020; Mehmood et al., 2021; Mekruksavanich & Jitpattanakul, 2021).

In light of this, the current study aims to compound the technical knowledge acquired in both human and non-human domains in order to establish automated bird behavior classification techniques. We used DeepLabCut (DLC: Mathis et al., 2018) as a markerless pose estimation tool to procure multivariate time series data. As a further step, we developed a module named Winkie (a name which was inspired by a Dickin Medal owning pigeon of the same name that had assisted in the rescue of an aircrew during the Second World War). This module consists of submodules for pre-processing and normalizing of the DLC data. Afterward, we applied and compared different machine learning and deep learning architectures to classify pigeon behaviors like eating, standing, walking, head shaking, tail shaking, preening, and fluffing. As a machine learning architecture, random forest gave a high weighted F1 score (0.81) over all behaviors and showed good performance for behaviors that were stable along spatial and temporal dimensions (such as eating, fluffing, preening, standing). The deep learning architecture InceptionTime, as a one-dimensional convolutional neural network (CNN), also demonstrated high overall performance (0.87). However, the particular performance for the highly dynamic behaviors such as head shake and tail shake were increased substantially in comparison to random forest.

## Method

**Fig. 1:**
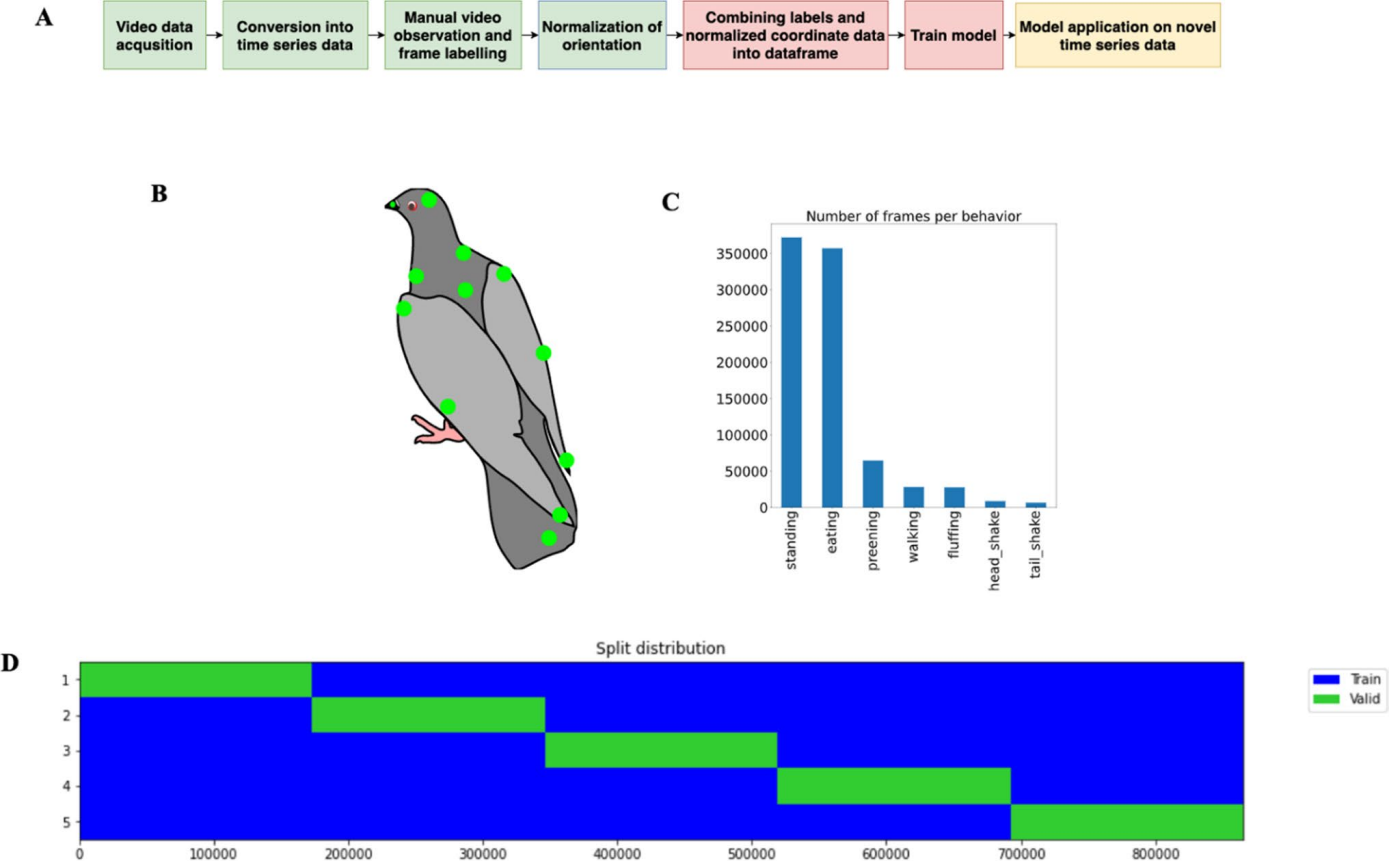
Data preparation. **A** The Winkie module consists of a sequential multi-step process for pigeons. **B** The tracked body points (head, beak, left-right neck, body, left up-middle-down wing, right up-middle-down wing, tail). **C** The number of frames per behaviors extracted after applying LabelAssistant. **D** The split distribution of fivefold non-shuffle cross-validation

Using the Winkie module as part of a research workflow consists of a sequential multi-step process as shown in Fig. 1A. The same process is used for evaluating its performance itself and each step will be discussed in the further sections.

## Data acquisition and manual observation

Eight naïve adult homing pigeons (*Columba livia*) from local breeders were maintained at 85–90% of their free-feeding body weight throughout the experiment, while water was accessible ad libitum. The experiment was conducted in a wooden box with a feeder located in the middle part. All procedures followed the German guidelines for the care and use of animals in science and were in accordance with the European Communities Council Directive 86/609/EEC concerning the care and use of animals for experimentation. They were also approved by Ruhr University Bochum, Germany.

Depending on their activity level in the experiment, pigeons received between 10 and 20 (10 min each) sessions in which they could freely move and consume grains. For instance, while highly active individuals were trained for 20 sessions to increase the possibility of dynamic behavior occurrence, the stabile individuals received ten sessions. Each session was recorded with a GoPro HERO7 at 119.88 frames per second with resolution of 1280 x 960 pixels. Initially, the videos were manually checked in detail to detect any occurrence of individual behaviors as described below and shown in Supplementary Video1:

- *Eating:* Consuming the food in the feeder or food dropped on the experiment platform.
- *Standing:* Remaining at the same location for an indeterminate period.
- *Walking:* Changing the location.
- *Head Shaking*: Moving the head along a curve in fluent and repeated motion.
- *Tail Shaking:* Moving the tail along a curve in fluent and repeated motion.
- *Preening:* Maintenance behavior that involves the use of the beak to reposition feathers on different parts of the body. A preening event started each time when the beak touched the body and finished once the beak lost contact with the body.
- *Fluffing:* Partial or total extension of one or both wings and ruffling feathers. Additional flapping of wings might occasionally occur.

The observer noted the time slice (starting and ending timecode) in which the behaviors occurred.

## Pre-processing video data

### Markerless pose estimation and manual observation verification

Video-tracking was performed using the machine-learning-based tracking software DeepLabCut (DLC: Mathis et al., 2018). Since we were interested in behaviors in which different body parts are actively involved, we tracked different points from the pigeon body as shown in Fig. 1B (head, beak, left-right neck, body, left up-middle-down wing, right up-middle-down wing, tail). The data acquired via DLC processing consist of multivariate time series data, which is a series of location values per body part over a period of time. For instance, a 10-min video recording results in approximately 71,928 frames (600 s x 119.88 fps). In this sense, our usage of DLC can be understood as a lossfull, but semantically enriched and transformed, data reduction step: For each frame, we go from raw (decompressed) 1280 x 960 x 8 bit = 9.5 MBit to 10-body parts x 2 x 32 bit = 640 bit. This comes down to a reduction factor on the bandwidth of the input data of roughly 15,000. In total, this process generated multivariate time series data for 10,424,241 frames.

In order to ensure the feasibility of detecting behaviors exclusively from the multivariate time series data, it was necessary to check whether a human observer can identify the behaviors mentioned above on the DLC as well. Therefore, we developed a module called *Pigeon Animator* for visualization of time slices with frame precision. In addition to verifying the behaviors and performance of the tracking, we narrowed the time slices by replacing the timecodes with frame numbers (see Supplementary Video2).

### Labeling

After applying the verified labels of the manual observer to the individual frame data, non-labeled frames were removed from the data set, which led to 865,548 remaining labeled frames. In order to make this process less error-prone, we developed a custom module *LabelAssistant* that would ensure the integrity of the labels with regards to the DLC output and safeguard against specific error classes (e.g., ensuring consistency in the label names). As shown in Fig. 1C, we ended up with an imbalanced data set, especially since the natural frequency of occurrence of the behaviors is already unbalanced. The challenge of class imbalance will be discussed in the following sections in more detail.

### Transformation and normalization of two-dimensional Euclidean input data

DLC tracks absolute coordinates, while the behavior should be considered by looking at the data from a relative standpoint in order to simplify classification and pattern matching (e.g., for hand gesture analysis: Do et al., 2020). In DLCImporter, we developed parameterizable functions for pre-processing the DLC data by normalizing it such that the body is translated into the origin (and the other parts translated accordingly, thereby representing the relative position to the body) while rotating all points such that the vector between body and the middle of the neck (basically the “spine”) becomes parallel to the *y*-axis. Since the body is in the origin, the spine is thereby implicitly located along the *y*-axis.

For each frame, the displacement vector *s* is defined as:

1 $$s=\left(- coordinate\left( body,x\right),- coordinate\left( body,y\right)\right)$$

and each body part *bp* is translated using *s* as the translation vector in the translation function T_v_:

2 $${T}_V(bp)= bp+s$$

In addition, a new body part *middle neck* is added which for our data is defined as:

3 $${v}_{middle\ neck}={v}_{left\ neck}-{v}_{right\ neck}$$

Based on this vector, the necessary rotation *rot*_*norm*_ as the angle in degree between the positive *x*-axis and the vector v_middle neck_ is calculated:

4 $${rot}_{norm}=\mathit{\arctan}2\left({v}_{middle\ neck\ x},{v}_{middle\ neck\ y}\right)\times \frac{180}{\pi }$$

Using this angle, the rotation matrix *R*_*norm*_ was constructed and applied on all body parts:

5 $${R}_{norm}=\left[\begin{array}{cc}\cos {rot}_{norm}& -\sin {rot}_{norm}\\ {}\sin {rot}_{norm}& \cos {rot}_{norm}\end{array}\right]$$

## Machine learning and deep learning architectures for behavioral classification

As discussed in the Introduction section, there is a plethora of machine learning and deep learning architectures that can be used to classify human activity and behaviors, each with its own strength and weaknesses for specific permutations of domains and input data. In addition, the field is constantly evolving, with new architectures and improved methods emerging continuously. In order to demonstrate the general feasibility of our approach, we selected *random forests* as a machine learning architecture and *InceptionTime* as a deep learning architecture.

The raw features that were used in both *random forest* and *InceptionTime* were defined according to their tracking likelihood values as given by DLC, which was an indication of the overall stability of the tracked body point. Accordingly, the *x* and *y* pixel coordinates of ‘head, left-right neck, body, left up-middle-down wing, right up-middle-down wing, tail’ were used as features, while ‘beak’ was excluded since tracking was unstable due to the frequent occlusion of the beak by the pigeon itself.

Overall generalization performance of fitted models is measured using five-fold non-shuffled cross-validation-score (Fig. 1D) as the arithmetic mean (10) of the weighted F1 score (9), to cater for imbalances of classes in the data set. F1 score (8) is an established scoring mechanism for measuring the accuracy of an information retrieval system and is defined as the harmonic mean of precision (6) and recall (7) (Rijsbergen, 1979; Chinchor, 1992). Although most of the literature studies have opted to shuffle data as part of the train-test split, due to the time series nature of the data at hand containing implicit dependencies between consecutive data points, we decided against it, since this would lead to unrealistically good test scores (since very similar data can end up in the train- and test-set). This problem is further amplified by the fact that the original video recordings were performed using a high framerate of 119.8 FPS. In addition, the last fold (first 80% as training-set and last 20% as test-set) was used to show the classification performance of individual behaviors.

6 $$precision=\frac{True\ Positive}{True\ Positive+ False\ Positive}$$

7 $$recall=\frac{True\ Positive}{True\ Positive+ False\ Negative}$$

8 $${F}_1=\frac{2}{precision^{-1}+{recall}^{-1}}$$

9 $$F{1}_{weighted}=\frac{1}{total\_ targets}\times \sum_{i=1}^{n\_ classes}F{1}_i\times {targets}_i$$

10 $${cv}_5=\frac{1}{5}\left(\sum_{i=1}^5F{1}_{weighted}(i)\right)$$

All evaluations were performed using an AMD Ryzen 9 5950X @ 3.4–4.9 GHz, 32GB RAM, NVIDIA GeForce RTX 2070 Super 8GB RAM, running on Microsoft Windows 10 Pro Build 19043. The Python machine-learning library scikit-learn (Pedregosa et al., 2011) was used for the random forest classifier and overall performance metrics while the deep learning stack of tsai, fast.ai and PyTorch was used for InceptionTime (Paszke et al., 2019; Oguiza, 2020; Howard & Gugger, 2020).

### Decision Trees and Ensemble Methods (Random Forest)

A decision tree in the context of machine learning can be understood as a binary tree with each node in the tree splitting the source set based on an inferred criteria of an input feature, leading to leaves containing the resulting class or a specific probability distribution of the classes. Decision trees used as classification trees have been shown to be an intuitive way to classify and label objects and if they are trained on high-quality data, they ensure very accurate predictions (Caruana & Niculescu-Mizil, 2006; Kingsford & Salzberg, 2008). The intuitive character of decision trees can be demonstrated by giving an example of how a human might intuitively build one: For example, if you want to construct a decision tree to identify the owner of a chirping sound, you can narrow down the possible answers by asking several consecutive and potentially dependent questions for binary splitting: Which birds are abundant during the current season, which ones are songbirds, is it night or day, etc. Each question will narrow the options and you will go on asking these questions until you reach a highly certain answer. Depending on the data used as input (the features that can be extracted from this input) and the possible answers to a sequence of questions (nodes), the resulting leaf node might contain a clear-cut answer (it is a Jay), or a distribution of class predictions (70% Kookaburra, 30% Lyrebird).

To further improve the predictive performance of machine learning algorithms, ensemble learning can be used to combine multiple different models into a single model (Dietterich, 2000; Peterson & Martinez, 2005). Random forests are an ensemble learning method for classification that makes use of a set of different decision trees and is shown to generally outperform decision trees (Ho, 1995; Piryonesi & El-Diraby, 2020).

For our model, the number of maximum features per split *max*_*f*_ was defined as:

11 $${\mathit{\max}}_f= sqrt\left({n}_{features}\right)$$

A good hyperparameter value n_estimators_ for the number of trees in the forest was determined by calculating the validation curve for the set s_estimators_:

12 $${s}_{estimators}=\left\{x\in \mathbb{N}\ |\ 1\le x\le 100\right\}$$

A reasonable number of trees considering the tradeoff between accuracy and time efficiency was selected as the hyperparameter to detect the performance of individual behaviors. In addition, the learning curves for different training set sizes were evaluated to determine the correlation between training set size, classification performance, and training time.

The model was created by segmenting the time series data into windows of different sizes using a sliding window approach with a step size of 1. The effect of different sizes on the performance was evaluated using a validation curve with the number of consecutive frames included in the input vector as a hyperparameter:

13 $${\mathit{\dim}}_{v_{input}}=\left({n}_{features}\times {window}_{size},1\right)$$

In order to combat the class imbalance, to which decision trees are sensitive, all models were trained using balanced class weights, with the weight *w*_*c*_ for a class *c* adjusted to be inversely proportional to class frequencies in the input data (Sun et al., 2009):

14 $${w}_c=\frac{total\ number\ of\ observations}{number\ of\ classes\times {number\ of\ observations}_c\ }$$

### InceptionTime

Through *AlexNet* winning the visual recognition challenge competition (*ImageNet*) in 2012, deep CNNs have been established as a state-of-the-art technique for domains such as image recognition, object detection or natural language processing, often reaching human levels of performance (Ren et al., 2015, Fawaz et al., 2019). Accordingly, Fawaz et al. (2020) propose *InceptionTime* to be an *AlexNet* equivalent for time series classification, in which an ensemble of deep CNN models (inception modules) is used for classification of multivariate time series data.

The optimal depth of the network depends on the lengths of patterns contained in each time series segment. In order to evaluate the effect of the depth hyperparameter on the model performance, we calculated the validation curve for the parameter range 1–6, with 6 being the default for *InceptionTime*.

Similar to the random forest, the time series was segmented using a sliding window approach with step size of 1. The window size was kept at 16 frames, which seemed suitable to capture not only long patterns, but also sudden and short ones.

The fitting of the models was done using one-cycle super-convergence training for learning rate adaption as dynamic hyperparameter tuning (Smith & Topin, 2018). Mock training with cyclical learning rates was used to determine a good maximum learning rate (Smith, 2017), with the steepest point of the resulting learning rate curve being selected as the maximum learning rate.

According to Smith (2018), although historically small batch sizes have been recommended for regularization effects, when applying a one-cycle learning rate schedule (as we do) a high batch size can be used to minimize computational time, while still achieving high performance. With regards to our available GPU memory, a batch size of 1024 was selected.

## Post processing

Applying any of the aforementioned models (on novel or existing data) returns a probability vector x_p_ with dim(x_p_) = n_classes_, where n_classes_ is the total of different classes, for each classified frame, respectively each classified time window. In addition, the sum of all vector elements is always equal to 1. Conservatively, applying:

15 $$b(f)= argmax\left({x}_p(f)\right)$$

will yield the predicted behavior *b* at frame *f*.

For binary classification models, traditionally different threshold values for selecting a prediction (compared to 0.5) can be applied to further tune the results with regards to precision and recall, depending on the needs of the application (Fielding & Bell, 1997). Inspired by these approaches, we propose an algorithm that allows individual thresholds for behavior tuples in a multi-class model:

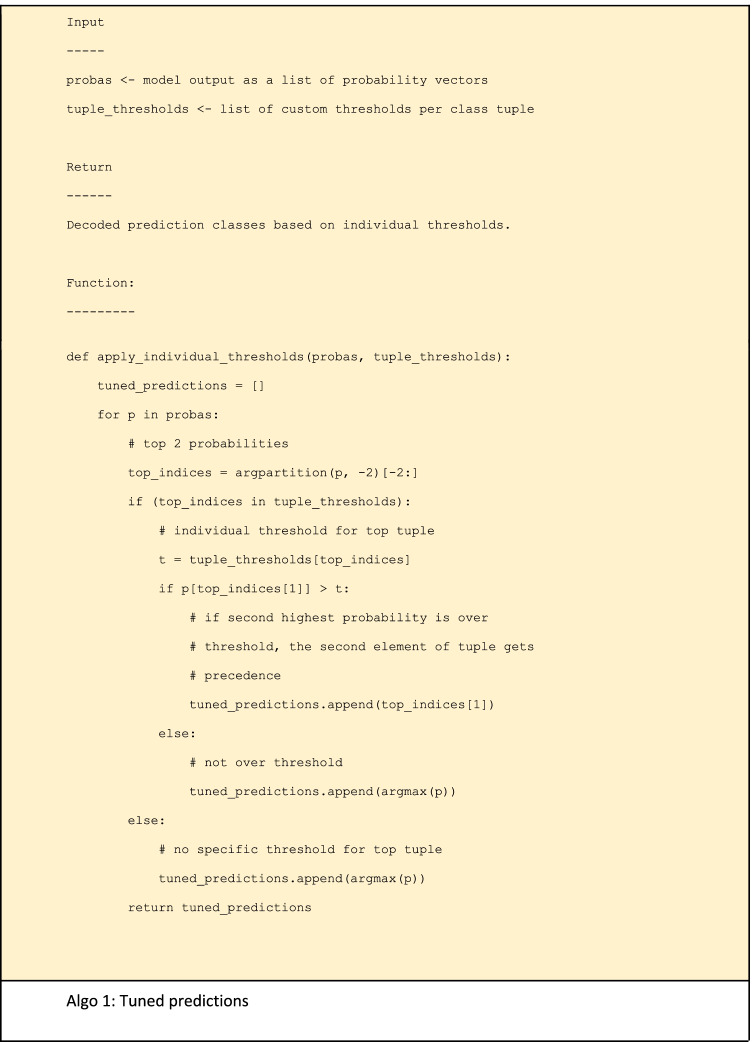



It is up to the user how those tuples are defined or optimized. However, we will show the effect of some a-posteriori chosen example values in the results section.

## Results

### Animal tracking

DLC training was performed using 1,030,000 iterations, achieving a root mean square error (RMSE) over all tracked body parts of 2.53 pixels for the train set and 6.41 pixels for the test set. Using a prediction cutoff value of 0.6, the train error remained the same and the test error could be reduced to 6.16 pixels. For our given video resolution of 1280 x 960 pixels, this translates to roughly 5.6 mm in the physical world.

### Random forest performance

**Fig. 2:**
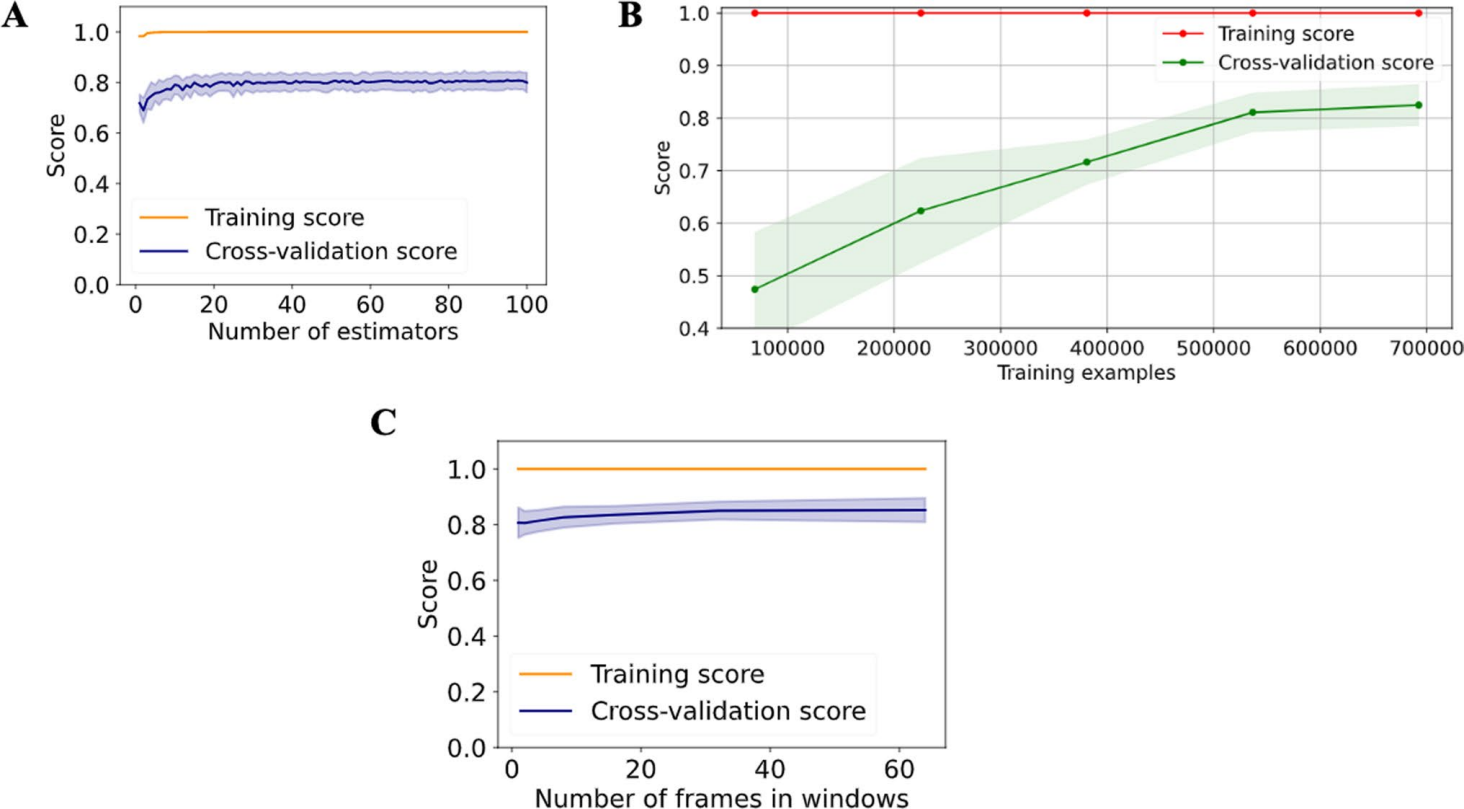
Random forest evaluation. **A** Validation curve for random forest for number of trees as hyperparameter. No substantial improvement of score for n>20. **B** Learning curve and performance for different amounts of training examples. **C** Validation curve for different window sizes

**Fig. 3:**
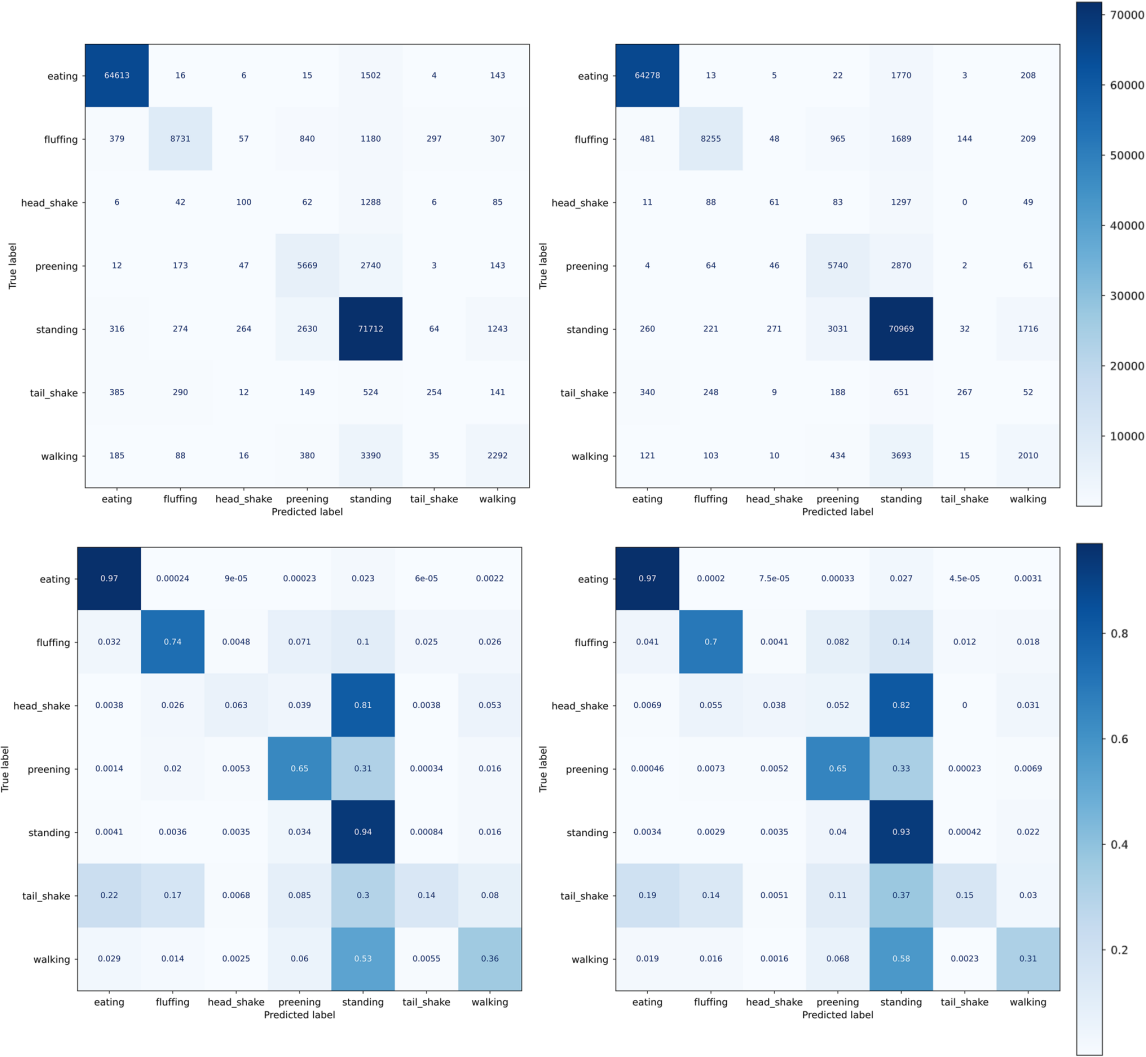
Confusion matrix for random forest. Confusion matrix for random forest with single frame (left) and sixteen frames (right) windows size (top absolute, bottom relative)

The validation curve for the n_estimators_ hyperparameter was calculated and analyzed, revealing a sufficiently good cross-validation score of 0.79 for 20 trees, with the maximum score of 0.81 occurring for 95 trees (Fig. 2A). Based on this finding, the learning curve for 20 trees was calculated, showing a continuous increase of the cross-validation score as a function of training-set size. However, the learning curve seems to reach a saturation point, for the maximum amount of available training data in our case (Fig. 2B). Further window size evaluation using a validation curve revealed that the overall performance was not strongly affected by the size of the window (Fig. 2C: F1_single frame_ = 0.807 ± 0.054, F1_2 frames_ = 0.806 ± 0.042, F1_4 frames_ = 0.813 ± 0.038, F1_8 frames_ = 0.827 ± 0.038, F1_16 frames_ = 0.835 ± 0.031, F1_32 frames_ = 0.850 ± 0.031, F1_64 frames_ = 0.852 ± 0.043). Since sudden behaviors occurred in short bursts of roughly 16 frames, we further compared the single frame and 16 frames performance in detail for individual classes as shown in Fig. 3. Both models gave high classification performance for the behaviors that were stable along spatial and temporal dimensions (meaning the behavior can be accurately classified by assessing the posture in a single frame). The individual behaviors’ classification performance remained mostly similar, except for preening and walking. While preening detection was slightly increased for 16 frames, walking detection was slightly decreased. Note that our transformation and normalization steps on the input data remove characteristics of the walking movement, since the coordinates are transformed into a more stable position.

### InceptionTime performance

**Fig. 4:**
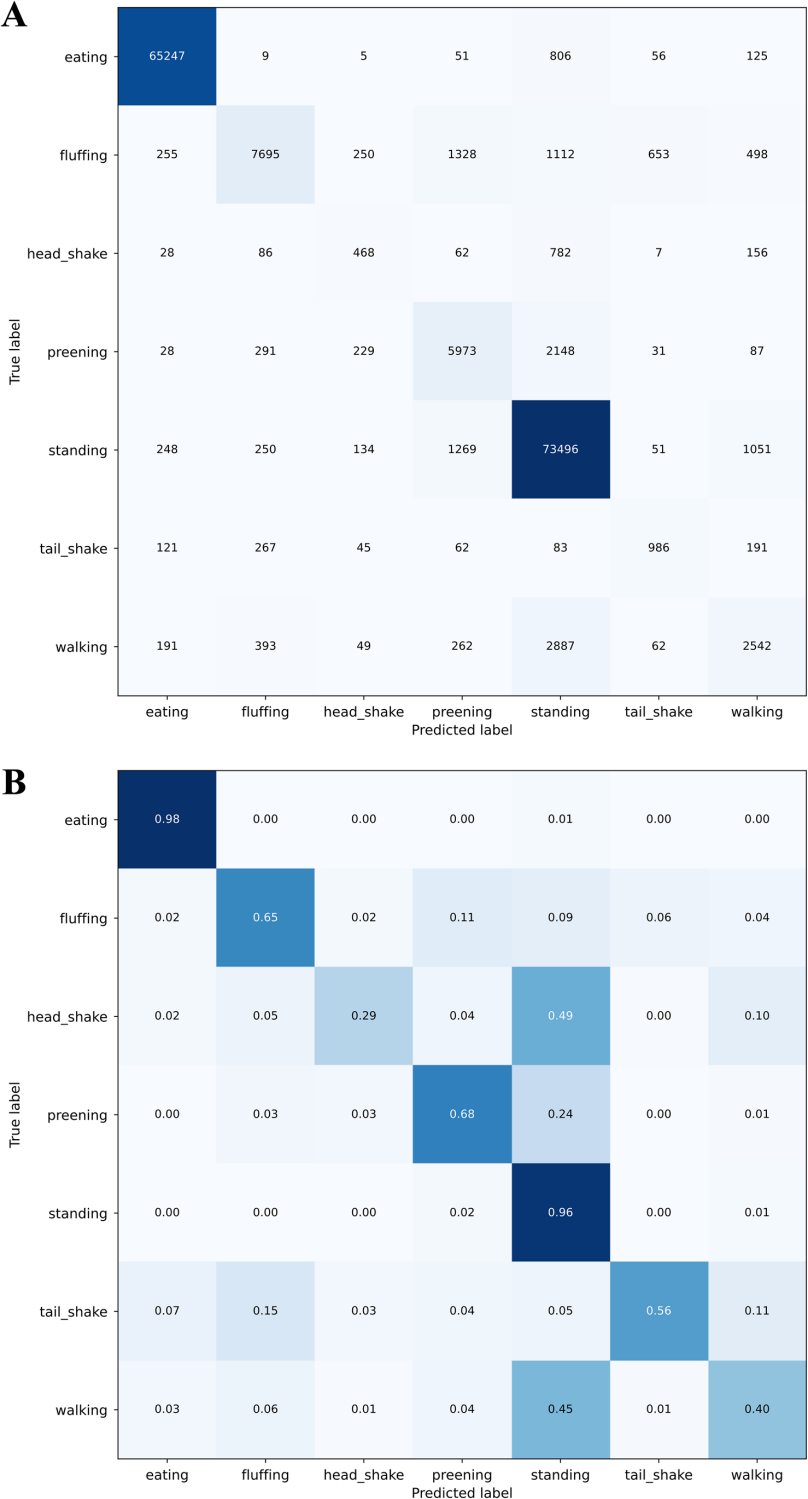
Confusion matrix for InceptionTime. **A** Confusion matrix for InceptionTime with absolute values. **B** Confusion matrix for InceptionTime with relative values

The validation curve for the depth hyperparameter indicated no significant effect of depth on the generalization performance as seen in the cross-validation score (Supplementary Figure 1). In order to reduce the complexity of the model and reduce the potential for overfitting, the smallest depth value of 1 with a F1 cross-validation score of 0.874 ± 0.031 (which was higher than the best scores achieved using *random forest*) was selected for the further evaluation. By calculating the confusion matrix on the last fold, similarly to *random forest* a good performance was acquired for behaviors that were stable along spatial and temporal dimensions. In addition, an increase in performance, compared to *random forest*, was also achieved on highly dynamic behaviors such as head shake and tail shake (Fig. 4A and Fig. 4B) (recall_head shake_ 0.064 vs. 0.36 and recall_tail shake_ 0.16 vs. 0.54).

### Post processing and model application

When analyzing the *InceptionTime* confusion matrix, we observed a prevalent confusion between ‘head shake - standing’ and ‘preening - standing’. Based on this observation, we defined the dynamic tuple thresholds as follows: [(standing, head shake): 0.2, (standing, preening): 0.1]

This changed the predictions in favor of precision (head shake increased to 0.63 and preening increased to 0.78) but led to worse recall (head_shake dropped to 0.08 and preening dropped to 0.57). Therefore, the tuple threshold needs to be adjusted to personal needs, e.g., is it more important to not miss any potential behaviors, or to reduce the number of false positives?

**Fig. 5:**
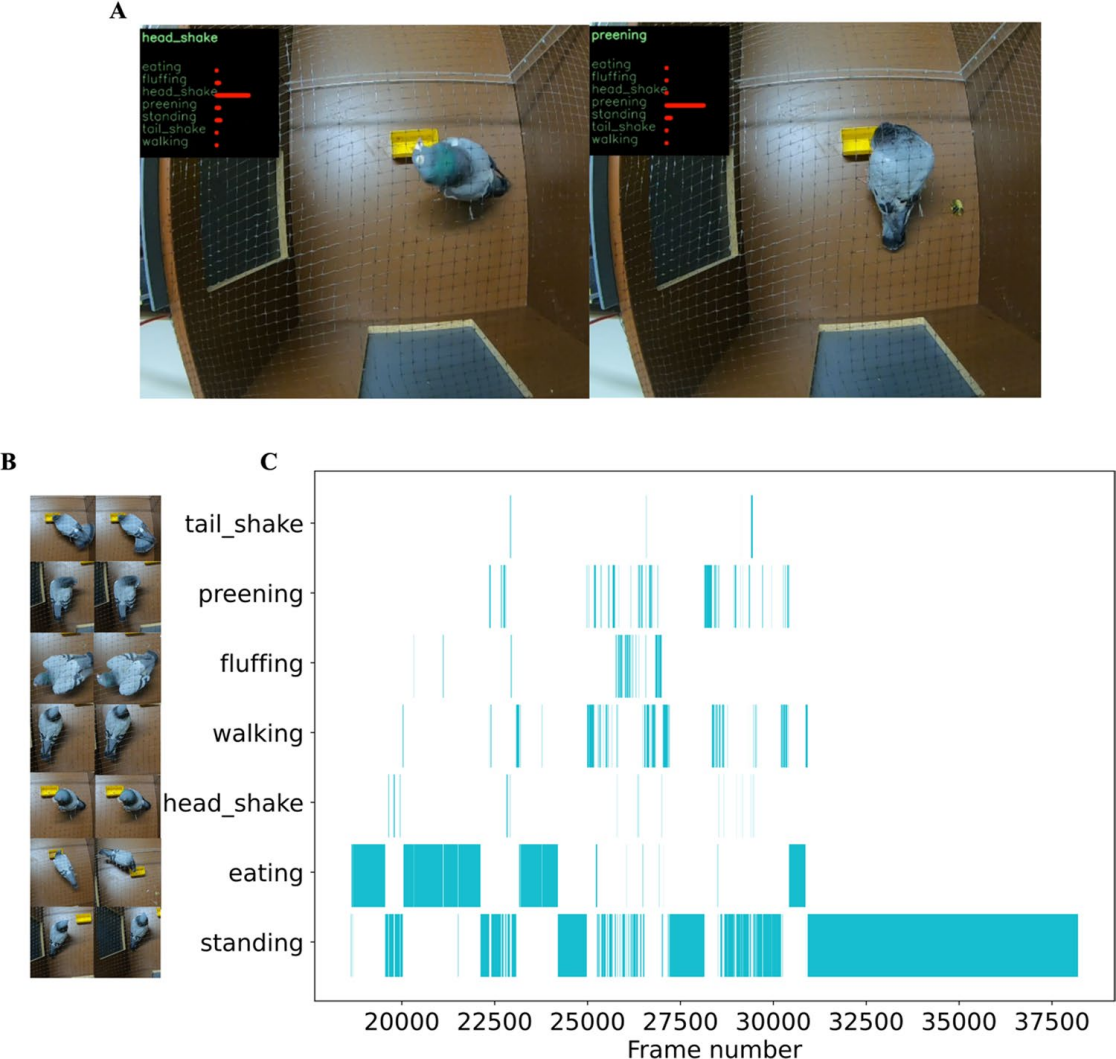
Classification results on novel video. **A** One possibility to evaluate the model performance is applying the model to the new data to get predictions and render the original videos with the predictions as overlay. **B** Behaviors that were checked in the videos. **C** Ethogram of predictions for novel data. Observer can go to the related frame number in the original or rendered video to double check the occurrence of the behavior

There are different possibilities to evaluate the output of the model for new data. While it is possible to directly work with the model output in a quantitative way, it seems desirable to also acquire forms of visualization that lend itself better to some form of human “quality control”. It is therefore possible to render the original videos with applied predictions (Fig. 5A, Supplementary Video3) or visualize the predicted behaviors over time in the form of an ethogram (Fig. 5B). Both techniques can also be effectively used in conjunction. By assessing the ethogram, a user is able to gather a general overview of the occurring behaviors at a specific point in time at a quick glance. Interesting (or suspicious) looking predictions can be counter-checked using the rendered videos containing the predictions as a text overlay. Especially in combination with tuned tuple thresholds, this can lead to a process that, while not fully automated, significantly augments the previous manual and laborious process.

### Discussion

We have demonstrated the feasibility of adopting existing machine learning classification approaches for pigeon behavior by using a simple single camera setup without further tracking equipment. To further improve the usability of our approach, we have developed and released the open-source software library Winkie to act as a starting point for future improvements and developments. Winkie is usable with commercial off-the-shelf computer hardware. While it might be possible to perform the classification directly on the video streams (Bohnslav et al., 2020), our software uses multivariate time series data as created by DLC to reduce the size and complexity of the video input data. Therefore, our software positions itself inside an ecosystem of emerging de facto industry standards of the scientific open-source community. Furthermore, users can configure the software depending on their needs, to change its bias between precision and recall for specific pairs of behaviors.

Although there is a movement among passionate psychologists and neuroscientists to augment their experimental paradigms with automated behavioral tracking (Lochmatter et al., 2008; Crispim Junior et al., 2012; Kabra et al., 2013; Pérez-Escudero et al., 2014; Mathis et al., 2018; Bello-Arroyo et al., 2018; Yamanaka & Takeuchi, 2018; Sridhar et al., 2019), existing automation tools generally lack support for analyzing bird-specific behaviors. Besides leading to significant amounts of time savings, our software aimed to extend the species-specific knowledge as a catalyst towards the initiation of the classification of bird behavior and to inspire new approaches (Miller, 1988). Considering the challenges to combine behavioral and neurophysiological measurements, we focused on developing a software that can be used with raw behavioral data being captured from simple hardware setups, such as single camera video recordings.

The relevance of our approach becomes visible when concentrating for example on behaviors like preening, scratching, or head shaking, which are typical avian maintenance and comfort actions (Cotgreave & Clayton, 1994). However, encountering a stressful situation induced by a competitor or predator can elevate the occurrence of these activity patterns (Delius, 1967, 1988; Fernández-Juricic et al., 2004; Wittek et al., 2021). Similarly, preening rates also increase after injections of dopamine or adrenocorticotropic hormone, with the latter also showing increased head shaking (Delius et al., 1976; Delius, 1988; Acerbo, 2001; Kralj-Fiser et al., 2010). Thus, these actions can serve as a behavioral readout of social conflicts and/or neural processes. But although a vast variety of research has reported these behaviors (Miller, 1988; Moyer et al., 2003; Prior et al., 2008; Clary & Kelly, 2016; Kraft et al., 2017; Wittek et al., 2021), there has been no exact classification and automated analysis of them so far. By using our approach, it is easily possible to disambiguate and quantify different kinds of reactions of the animal along the time frame in stressful contexts and/or when injected with various drugs. Thus, we anticipate that this open-source library, and other developments inspired by it, will pave the way for a more quantitative behavioral analysis of different bird species and beyond.

### Future directions and challenges

In this manuscript, we demonstrated how machine learning systems can support classical experimental-psychological and ethological approaches by detecting and quantifying avian behavior. It is important to note that future studies should bear in mind that not only the amount but also the sequence of behavior contains highly relevant insights. Besides stereotypical behavioral patterns recorded by classical ethological approach, there are also subpatterns which might be of interest. This can be described with an ontology in which patterns are an aggregate of subpatterns. For example, head shake can be defined as a continuous and alternating sequence of the head-move-left and head-move-right subpatterns that can remain undetected by manual observation or supervised learning, simply because they are ignored or unknown (Luxem et al., 2020). One promising solution to detect such subpatterns and possible behavioral sequences is using unsupervised machine learning techniques. Besides, in order to understand the functional framework of these behavioral sequences, their correlation with neural activity patterns is easy to implement. In addition, for the behaviors that are hard to capture in two dimensions due to occlusions resulting from the camera perspective, three-dimensional tracking should be considered (Nath et al., 2019).

We have used the DLC output as-is, without applying the filters available in DLC or implementing our own. The lack of filtering might lead to glitches in tracking and anatomically impossible movements. Besides using the available DLC filters or other generic approaches for smoothing such as Kalman filtering (Kalman, 1960) or applying the Ramer–Douglas–Peucker algorithm (Wu & Marquez, 2003), tracking can be also smoothed and aliased by formulating anatomical constraints for the tracked skeleton through inverse and forward kinematics (Halvorsen et al., 2008; Nilsson et al., 2020).

Besides our multiclass classification approach, applications from the human domain have shown promising results when using fewer classes or ensemble classifiers with multiple binary classification models (Jethanandani et al., 2019), which possibly induces better performance. As we explained in the Method section, we ended up with an imbalanced data set in which rare behaviors like head shake and tail shake were not equally present. Although this fits the natural occurrence frequency of these behaviors, generating a more balanced dataset, by including more data of minority classes, undersampling of majority classes or by using synthetic oversampling techniques on the minority classes (such as SMOTE: Chawla et al., 2002), could lead to better performance for all variants of classification.

Overall, we demonstrated that existing machine learning approaches can be used in conjunction with markerless pose-estimation tracking data in pigeons – a classic laboratory animal in psychological research on learning, memory, and cognition. The trained model showed high performance on the validation data that was never seen by the model before. In addition, we developed an open-source library as a starting point for further automated classification of bird behaviors. Our system is interface compatible with other machine learning architectures from scikit-learn and PyTorch and is thereby naturally extensible. We are hopeful that our system will help other scientists to extract detailed behavioral data under all kinds of different experimental conditions.

## Supplementary Information

ESM 1 (DOCX 65 kb)
