# Supplementary material for: Supervised machine learning aided behavior classification in pigeons
Source: Behav Res Methods. 2022 Jun 14;55(4):1624–40. doi: 10.3758/s13428-022-01881-w (PMC10250476; doi:10.3758/s13428-022-01881-w)
Supplement: Supplementary file 1 — (DOCX 65 kb) [file 13428_2022_1881_MOESM1_ESM.docx]

**Supplementary Figure 1**

*InceptionTime evaluation*


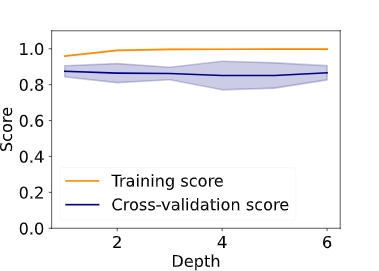


*Note.* Effect of depth-hyperparameter on training and cross-validation score.
